# Revision Surgery and Progression to Total Hip Arthroplasty After Surgical Correction of Femoroacetabular Impingement: A Systematic Review

**DOI:** 10.1177/03635465211011744

**Published:** 2021-06-03

**Authors:** Filippo Migliorini, Nicola Maffulli, Alice Baroncini, Jörg Eschweiler, Markus Tingart, Marcel Betsch

**Affiliations:** † Department of Orthopedics and Trauma Surgery, RWTH Aachen University Clinic, Aachen, Germany; ‡ Department of Medicine, Surgery and Dentistry, University of Salerno, Baronissi, Italy; § Queen Mary University of London, Barts and the London School of Medicine and Dentistry, Centre for Sports and Exercise Medicine, Mile End Hospital, London, UK; ‖ School of Pharmacy and Bioengineering, Faculty of Medicine, Keele University, Stoke on Trent, UK; ¶ Department of Orthopaedics and Trauma Surgery, University Medical Centre Mannheim of the University Heidelberg, Mannheim, Germany; Investigation performed at RWTH Aachen University, Aachen, Germany

**Keywords:** femoroacetabular impingement, revision surgery, total hip arthroplasty, risk factors

## Abstract

**Background:**

Femoroacetabular impingement (FAI) is a major cause of hip pain in young adults and athletes. Surgical treatment of FAI is recommended in cases of failed nonoperative treatment that have the typical clinical and radiographic findings. At present, the role of risk factors for revision surgery and progression to total hip arthroplasty (THA) in patients with FAI is still unclear.

**Purpose:**

To investigate the possible association between (1) rate of revision and progression to THA and (2) patient characteristics, type of lesion, family history of hip disease, type of intervention, radiographic parameters, physical examination, and pre- and postoperative scores.

**Study Design:**

Systematic review; Level of evidence, 4.

**Methods:**

The present systematic review was performed according to the PRISMA (Preferred Reporting Items for Systematic Reviews and Meta-Analyses) guidelines. In October 2020, the main online databases were accessed. All articles concerning surgical correction for selected patients with FAI were accessed. Patient characteristics, type of intervention, radiographic parameters, physical examination, and pre- and postoperative scores were assessed. The outcomes of interest were the possible association between these variables and the rate of revision and subsequent progression to THA using a multivariate analysis through the Pearson product-moment correlation coefficient.

**Results:**

Data from 99 studies (9357 procedures) were collected. The median follow-up was 30.9 months (interquartile range, 24.0-45.0). The mean ± SD age was 33.4 ± 9.3 years; mean body mass index (BMI), 24.8 ± 4.8; percentage right side, 55.8% ± 8.0%; and percentage female sex, 47.5% ± 20.4%. The overall rate of revision was 5.29% (351 of 6641 patients), while the rate of subsequent progression to THA was 3.78% (263 of 6966 patients). Labral debridement (*P* < .0001), preoperative acetabular index (*P* = .01), and BMI (*P* = .03) all showed evidence of a statistically positive association with increased rates of THA. No other statistically significant associations were found between patient characteristics, type of lesion, family history of hip disease, type of intervention, radiographic parameters, physical examination, or pre- and postoperative scores and the rate of revision and/or progression to THA.

**Conclusion:**

Although surgical procedures to treat FAI led to satisfactory outcomes, there was a revision rate of 5.29% in the 9357 procedures in the present systematic review. The rate of progression to THA after a median follow-up of 30 months was 3.78%. Patients who have a higher BMI and/or have a pathologic acetabular index and/or undergo labral debridement during correction of FAI are more at risk for a subsequent THA. We advocate additional education of this patient population in terms of expected outcomes and suggest surgical labral repair instead of debridement if needed.

In patients with femoroacetabular impingement (FAI), anatomic abnormalities of the femoral head and/or the acetabulum produce pathologically high contact forces between the femur and the acetabulum. FAI can be a cause of activity limitation, decreased hip function, and significant hip pain, especially in young adults and athletes, because of cartilage and labral damage.^38,39^ These repetitive insults to the cartilage and labrum result in early hip degeneration and osteoarthritis^106^; 79% of patients with osteoarthritis of the hip displayed subtle developmental changes on radiographs obtained before adulthood.^38,48^ FAI can be classified into 3 types depending on the origin of the pathology, being on the femur (cam), acetabulum (pincer), or both (mixed). In previous cross-sectional studies of 4151 individuals, 19.6% of men and 5.2% of women exhibited a pistol grip deformity of the proximal femur, which was defined by calculating the triangular index.^42^ Surgery is indicated in symptomatic patients with clinical and radiographic findings of FAI whose nonoperative treatment has failed for a minimum of 3 months.^62^ In these patients, surgical options include femoral osteochondroplasty to improve the femoral head-neck offset; debridement, repair, or reconstruction of the labrum; and/or removal of an excessive acetabular rim.^31,66,79^ Ganz et al^39^ first described the technique of surgical hip dislocation for the treatment of FAI in 2003, and several studies have shown good clinical outcomes using this technique.^5,91^ Given the long operating and recovery time of open hip dislocation surgery, a mini-open anterior technique was developed by Clohisy and McClure,^20^ who accessed the hip joint through a Hueter approach. Over the past few years, arthroscopic management of FAI has become popular, with a decrease in complications and faster recovery.^60,83,97^

All surgical interventions aim to improve patients’ activity levels, relieve hip pain, and restore natural hip function. The various surgical techniques for management of FAI are all successful (surgical hip dislocation, mini-open, arthroscopy), but data on rates of revision and progression to total hip arthroplasty (THA) are limited. So far, prognostic factors for surgical outcome for FAI are still unclear. Thus, the present systematic review investigated the risk factors for revision surgery and progression to THA in patients who underwent surgery for symptomatic FAI. A multivariate analysis was conducted to investigate the association between (1) rate of revision and progression to THA and (2) patient characteristics, type of lesion, type of intervention, radiographic parameters, physical examination, and pre- and postoperative scores.

## Methods

### Search Strategy

The present systematic review was performed according to the PRISMA (Preferred Reporting Items for Systematic Reviews and Meta-Analyses) guidelines.^77^ We followed the PICO protocol for the preliminary search:

*P (problem)*: FAI
*I (intervention)*: surgical correction
*C (comparator)*: generalities, type of intervention, radiographic parameters, tests, scores
*O (outcomes)*: revision rate and progression to THA

### Literature Search

Two authors (F.M., A.B.) independently performed the literature search in October 2020, accessing the following databases with no time constraints: PubMed, Embase, Google Scholar, and Scopus. The following keywords were used in combination: hip, FAI, femoroacetabular impingement, arthroscopy, mini-open, open, surgery, dislocation, treatment, therapy, cam, pincer, mixed, labral, acetabulum, femur, pelvis, pain, debridement, repair, reconstruction, THA, complications, pain. The resulting titles and eventually the abstracts were screened by the 2 authors. The full text of the articles of interest was accessed. The references were also screened. Disagreements between the authors were solved by a third senior author (M.B.).

### Eligibility Criteria

All the articles concerning surgical correction for patients with FAI were accessed. To be eligible for inclusion, articles had to report the rate of revision and/or progression to THA at last follow-up. Any kind of surgical intervention that did not involve THA was considered revision surgery. According to the authors language capabilities, articles in English, Italian, French, German, and Spanish were considered. Articles of level 1 to 4 according to the Oxford Centre of Evidenced-Based Medicine were considered.^55^ Data from national registries were not considered. Reviews, letters, expert opinion, case reports, and editorials were not eligible. Animal, biomechanical, and cadaveric studies were also not considered. Articles regarding revision settings were not eligible. Studies with data based on combined treatments, as well as those focusing on rehabilitation protocols, were excluded. Studies including adjuvants or innovative surgical procedures were excluded. The studies were included regardless of the surgical exposure (arthroscopic, mini-open, open). Studies treating skeletally immature patients were included, as were those describing outcomes in patients who were obese. Studies with data on patients >60 years old or with clear evidence of advanced hip degeneration (Tönnis grade III) were not included. Case series of <10 patients were also excluded. Only studies reporting quantitative data under the outcomes of interest were analyzed.

### Data Extraction

Data extraction was performed by 2 authors (F.M., A.B.). Data from the following endpoints were collected:

*Generalities*: author and publication year, journal, type of study, follow-up duration, number of patients and procedures, mean age, body mass index (BMI), sex, side of surgery, return to sport
*Type of intervention*: labral debridement, labral repair, labral reconstruction
*Radiographic parameters*: femoral offset (millimeters), acetabular inclination (Tönnis angle), α-angle (anteroposterior, groin-lateral), β-angle, sharp angle, center-edge angle, anterior center-edge angle, lateral center-edge angle, acetabular index, Tönnis grade, caudocranial femoral coverage (percentage), anterior coverage (percentage), posterior coverage (percentage), crossover sign, and joint space (medial, foveal, lateral)
*Physical examination*: range of motion (flexion, extension, abduction, adduction, internal and external); anterior, lateral, and posterior impingement test (percentage positive)
*Pre- and postoperative scores*: Harris Hip Score, modified Harris Hip Score, Non-arthritic Hip Score, 12-Item Short Form Health Survey (SF-12; physical and mental subscales), Hip Outcome Score (activities of daily living and sport-specific subscales), International Hip Outcome Tool–12 and −33, and visual analog scale

The present work investigated whether the aforementioned endpoints were associated with the rate of revision and subsequent progression to THA. Thus, every endpoint was independently analyzed, and its association with revision and progression to THA was assessed.

### Methodological Quality Assessment

The methodological quality assessment was made through the Coleman Methodology Score (CMS).^23^ The CMS analyzes studies under several items: number of patients, follow-up, type of surgical approach, and study design, as well as descriptions of diagnosis, surgical technique, and postoperative rehabilitation. Furthermore, outcome criteria, the procedure of assessing outcomes, and a description of the sample selection process are evaluated. The quality is scored from 0% (poor) to 100% (excellent), with values >60% considered satisfactory.

### Statistical Analysis

The statistical analyses were performed by the main author (F.M.). For the analytical statistics, STATA software (Version 16; StataCorp) was used. The Shapiro-Wilk test was performed to investigate data distribution. For normal data, mean and standard deviation were calculated. For nonparametric data, median and interquartile range were calculated. Multiple pairwise correlations using the Pearson product-moment correlation coefficient (*r*) were performed to investigate the association between the endpoints were accomplished. According to the Cauchy-Schwarz inequality, the final effect ranks between +1 (positive linear correlation) and −1 (negative linear correlation). Values of 0.1 < | *r* | < 0.3 and 0.3 < | *r* | < 0.5 and | *r* | > 0.5 were considered to have poor, moderate, and strong correlation, respectively. Possible associations with the outcomes of interest were evaluated for each endpoint. Overall significance was evaluated using the χ^2^ test. A linear regression of the statistically significant correlations was made, and added-variable plots were displayed. Values of *P* > .05 were considered statistically significant.

## Results

### Search Results

**Figure 1.**
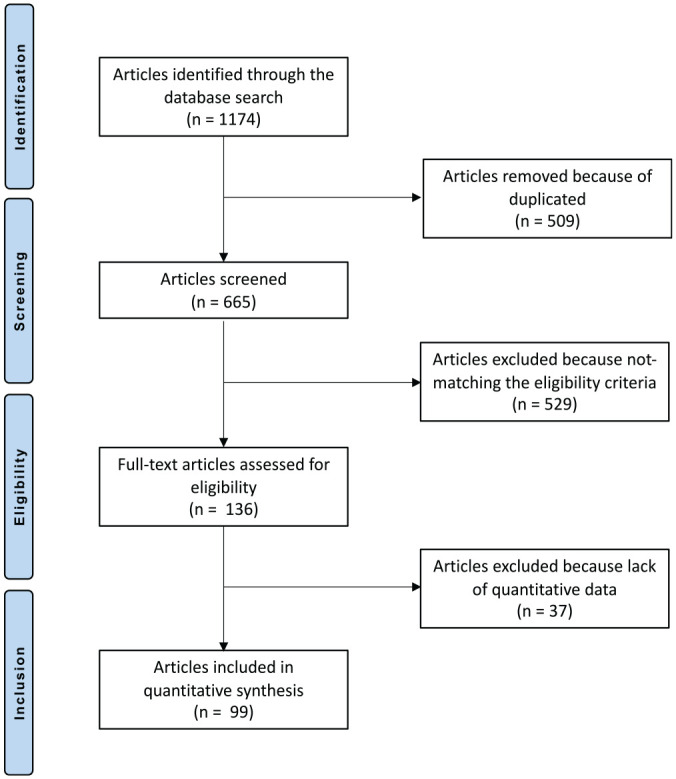
Flowchart of the literature search.

The literature search resulted in 1174 articles. Initially, 509 articles were excluded because of duplication; 529 articles were then excluded because of the following: type of study (n = 187), nonoperative techniques (n = 91), combined treatments (n = 47), adjuvants and/ or innovative surgeries (n = 41), language limitations (n = 22), uncertain data (n = 7), or other (n = 134). A further 37 articles were excluded because they did not match the topic of interest or report quantitative data under the outcomes of interest. Finally, 99 articles were included for analysis: 3 randomized clinical trials, 36 prospective studies, and 60 retrospective studies. The literature flowchart is shown in Figure 1.

### Methodological Quality Assessment

The CMS evidenced the overall limited quality of the studies. Indeed, 60% of studies were retrospective, 36% prospective, and only 3% were randomized trials. Eligibility criteria and rehabilitation protocols were frequently not indicated. General health measures were rarely cited. The procedure of assessing outcomes was often biased or not clearly described. The study size and mean follow-up were well-reported in most studies. The descriptions of diagnoses and surgical techniques were also commonly well-described. The overall CMS was 64.7 points (range, 40-85), attesting to the acceptable quality of the methodological assessment of the present study (Table 1).

**Table 1:** Generalities of the Included Studies and Demographic Baseline of the Patients^*a*^

First Author	Year	Journal	Study Design	CMS	Treatment	Mean Follow-up, mo	Procedures, No.	Mean Age, y	Female, %
Anwander^1^	2017	*Clin Orthop Rel Res*	Retrospective	68	Open	Resection, 156.0; reattachment, 144.0	60 (resection, 25; reattachment, 35)	Resection, 29; reattachment, 29	Resection, 24; reattachment, 37
Bardakos^2^	2008	*J Bone Joint Surg Br*	Retrospective	61	Arthroscopy	12.0	71	34.3	47.9
Beaulé^3^	2007	*J Bone Joint Surg Am*	Retrospective	54	Open	37.2	37	40.5	
Beck^4^	2011	*J Bone Joint Surg Am*	Retrospective	53	Open	12.0	50	32.0	44.0
Beck^5^	2004	*Clin Orthop Rel Res*	Retrospective	40	Open	56.4	19	36.0	
Bedi^6^	2011	*Am J Sports Med*	Prospective	53	Arthroscopy	10.9	10	25.9	
Bellotti^7^	2016	*Hip Int*	Retrospective	53	Mini-open	104.4	296		
Boone^8^	2012	*HSS J*	Retrospective	70	Open	46.8	22	44.0	31.8
Botser^9^	2014	*Am J Orthop*	Prospective	72	Arthroscopy	14.3	18	20.1	100.0
					Open	16.2	5	18.1	
Bryan^10^	2016	*Am J Sports Med*	Prospective	82	Arthroscopy	Younger, 51.6; older, 46.8	201	Younger, 37; older, 60	68.7
Büchler^11^	2013	*Arthroscopy*	Retrospective	61	Arthroscopy	15.0	66	33.8;	74.2
					Open	17.5	135	31.2	32.6
Byrd^12^	2009	*Clin Orthop Relat Res*	Prospective	79	Arthroscopy	16.0	207	33.0	31.0
Byrd^13^	2011	*Am J Sports Med*	Prospective	76	Arthroscopy	19.0	200	28.6	26.0
Byrd^15^	2009	*Arthroscopy*	Prospective	70	Arthroscopy	120.0	26	46.0	50.0
Byrd^16^	2016	*Arthroscopy*	Retrospective	70	Arthroscopy	30.0	244 (122, study; 122, control)	Study, 15.9; control: 36.8	Study, 53.3; control, 41.8
Camenzind^17^	2015	*J Hip Pres Surg*	Retrospective	66	Open	Reconstruction: 38.0; control: 42.0	27 (reconstruction, 13; control, 14)	Reconstruction, 36; control, 25	Reconstruction, 38.5; control 21.4
Chaudhary^18^	2015	*Indian J Orthop*	Retrospective	61	Open	24.8	16	28.3	31.3
Cho^19^	2015	*Hip Pelvis*	Retrospective	57	Open / mini-open	>24	13	45.0	53.8
Cohen^22^	2012	*Am J Sports Med*	Retrospective	59	Mini-open	22	66	32.0	31.8
Comba^25^	2016	*Muscles Ligaments Tendons J*	Prospective	72	Arthroscopy	91.0	42	38.0	37.7
Degen^26^	2017	*Arthroscopy*	Retrospective	66	Arthroscopy	Study, 36.1; control, 34.1	346 (study, 38; control, 306)	Study, 16; control, 31	Study, 47; control, 46.3
Domb^27^	2017	*Am J Sports Med*	Retrospective	68	Arthroscopy	Tönnis 0, 70.0; Tönnis 1, 72.6	124 (Tönnis 0, 62; Tönnis 1, 62)	Tönnis 0, 41.9; Tönnis 1, 42.3	59.7
Domb^28^	2014	*Am J Sports Med*	Retrospective	66	Arthroscopy	Reconstruction, 26.4; resection, 30.0	33 (11, reconstruction; 22, resection)	Reconstruction, 33.0; resection, 38.8	Reconstruction, 36.4; resection, 36.4
Domb^29^	2015	*Arthroscopy*	Prospective	77	Arthroscopy	Study, 32.8; control, 33.1	104 (study, 52; control, 52)	Study, 54.8; control, 20.3	65.4
Domb^30^	2013	*Arthroscopy*	Prospective	68	Open	24.8	10	19.0	80.0
					Arthroscopy	25.5	20	19.6	80.0
Espinosa^31^	2006	*J Bone Joint Surg Am*	Retrospective	61	Open	24.0	60	30.0	36.5
Ezechielia^32^	2016	*Technol Health Care*	Prospective	63	Mini-open	15.0	72 (group A, 56; group B, 15)	Group A, 32.1; group B, 28.5	47.2
Fabricant^33^	2015	*J Bone Joint Surg Am*	Retrospective	67	Arthroscopy	21.0	37	28.0	41.0
							149	30.0	50.0
							57	29.0	58.0
Flores^34^	2018	*Orthop J Sports Med*	Prospective	72	Arthroscopy	Early, 15.5; late, 13.1	60 (early, 30; late, 30)	Early, 37.2; late, 35.3	Early, 50.0; late, 43.3
Frank^35^	2014	*Am J Sports Med*	Retrospective	64	Arthroscopy	29.9	64 (partial capsular closure, 32; complete capsular closure, 32)	32.8	62.5
Fukui^36^	2015	*Arthroscopy*	Retrospective	75	Arthroscopy	40.0	102	35.0	50.0
Fukui^37^	2015	*Bone Joint J*	Retrospective	64	Arthroscopy	42.0	28	34.0	42.9
Gedouin^40^	2010	*Orthop Traum Surg Res*	Retrospective	58	Arthroscopy	15.6	38	36.0	13.2
Gicquel^41^	2014	*Orthop Traum Surg Res*	Prospective	64	Arthroscopy	55.2	53	31.0	62.7
Gupta^44^	2016	*Am J Sports Med*	Prospective	77	Arthroscopy	28.9	595	38.04	61.7
Gupta^45^	2014	*Am J Sports Med*	Prospective	71	Arthroscopy	28.3	47	37.18	40.4
Haefeli^46^	2017	*Clin Orthop Relat Res*	Retrospective	72	Arthroscopy	84.0	52	35.0	89.0
Hartigan^49^	2017	*J Hip Pres Surg*	Retrospective	64	Arthroscopy	42.0	69	43.6	36.9
Hartmann^50^	2009	*Arch Orthop Trauma Surg*	Retrospective	56	Arthroscopy	15.0	34	31.1	48.5
Hatakeyama^51^	2018	*Am J Sports Med*	Retrospective	69	Arthroscopy	42.5	45	Success, 20; failure, 47	Success, 59; failure, 91
Honda^52^	2020	*Knee Surg Sports Traumatol Arthrosc*	Retrospective	66	Arthroscopy	Young, 31.8; middle, 30.9	84	Younger, 30.9; middle, 56.7	Young, 46; middle, 67
Horisberger^53^	2010	*Arthroscopy*	Prospective	56	Arthroscopy	36.0	20	47.3	20.0
Horisberger^54^	2010	*Clin Orthop Relat Res*	Prospective	69	Arthroscopy	27.6	105	40.9	30.5
Hufeland^56^	2016	*Arch Orthop Trauma Surg*	Retrospective	64	Arthroscopy	66.3	44	34.3	45.5
Ilizaliturri^57^	2007	*J Bone Joint Surg Br*	Prospective	61	Arthroscopy	30.0	14	30.6	53.8
Ilizaliturri^58^	2008	*J Arthroplasty*	Prospective	60	Arthroscopy	24.0	19	34.0	42.1
Krych^61^	2013	*Arthroscopy*	Prospective, Randomized	76	Arthroscopy	32.0	36	Repair, 38; debridement, 39	100.0
LaFrance^63^	2015	*J Hip Pres Surg*	Prospective	71	Arthroscopy	PRP, 18.5; control, 23.3	35	PRP, 34.4; control, 34.9	
Larson^64^	2009	*Arthroscopy*	Retrospective	63	Arthroscopy	Group 1, 21.4; group 2, 16.5	71 (group 1, 36; group 2, 39)	Group 1, 31; group 2, 27	Group 1, 26.5; group 2, 37.8
Larson^65^	2012	*Am J Sports Med*	Prospective	79	Arthroscopy	42.0	94	Group 1, 32; group 2, 28	Group 1, 38.6; group 2, 42
Levy^67^	2017	*Am J Sports Med*	Retrospective	49	Arthroscopy	31.2	84 (atypical, 28; typical, 56)	Atypical 35.8; typical 35.2	Atypical 64; typical 64
Levy^68^	2017	*Am J Sports Med*	Retrospective	67	Arthroscopy	24.0	51	26.3	56.7
Maldonado^69^	2018	*Am J Sports Med*	Retrospective	65	Arthroscopy	IFL, 42.5; control, 43.9	743 (IFL, 351; control, 392)	IFL, 27.8; control, 34.1	IFL, 82.3; control, 70.7
Maldonado^70^	2019	*Arthroscopy*	Retrospective	57	Arthroscopy	CLT, 59.7; control, 51.4	72 (CLT, 18; control, 54)	CLT, 41.2; control, 41.1	50.0
Mardones^72^	2016	*Muscles Ligaments Tendons J*	Retrospective	61	Arthroscopy	48.0	17	33.5	73.3
Matsuda^73^	2015	*J Hip Pres Surg*	Prospective	69	Arthroscopy	>24	145 (focal, 127; global, 18)	Focal, 39.8; global, 37.2	Focal 52; global, 33
McConkey^74^	2019	*J Pediatr Orthop*	Prospective	60	Arthroscopy	24.0	36 (bilateral, 24; unilateral, 12)	Bilateral, 15.7; unilateral, 16.5	Bilateral, 58.3; unilateral, 58.3
Mohan^76^	2017	*Arthroscopy*	Retrospective	69	Arthroscopy	34.0	57	17.8	66.0
Moriya^78^	2017	*J Orthop Surg Res*	Retrospective	61	Arthroscopy	28.0	23	59.3	73.9
Murphy^79^	2004	*Clin Orthop Relat Res*	Prospective	71	Open	62.4	23	35.4	43.5
Naal^80^	2012	*Am J Sports Med*	Retrospective	68	Open	60.7	233	30.0	40.0
Naal^81^	2011	*Am J Sports Med*	Retrospective	59	Open	45.1	30	19.7	0.0
Nawabi^82^	2016	*Am J Sports Med*	Prospective	62	Arthroscopy	31.3	207 (BD, 55; control, 152)	BD, 29.8; control, 29.6	BD, 47.8; control, 55.7
Nho^83^	2011	*Am J Sports Med*	Retrospective	61	Arthroscopy	27.0	47	22.8	28.0
Nielsen^84^	2014	*BMC Musc Dis*	Prospective	72	Arthroscopy	>24	117	37.0	59.0
Novais^85^	2014	*J Pediatr Orthop*	Retrospective	41	Open	21.6	29	17.0	31.0
Palmer^87^	2012	*Arthroscopy*	Retrospective	72	Arthroscopy	46.0	185	40.2	50.7
Perets^88^	2017	*Arthroscopy*	Prospective	67	Arthroscopy	35.7	11	14.7	100.0
Perets^89^	2018	*Arthroscopy*	Retrospective	62	Arthroscopy	49.1	60	19.5	80.0
Perets^90^	2018	*J Bone Joint Surg Am*	Retrospective	68	Arthroscopy	Obese, 71.6; control, 71.3	148 (obese, 74; control, 74)	44.2	Obese, 60.8; control, 60.8
Peters^91^	2006	*J Bone Joint Surg Am*	Prospective	60	Open	32.0	30	31.0	44.8
Peters^92^	2010	*Clin Orthop Relat Res*	Retrospective	58	Open	26.0	96	28.0	41.5
Philippon^93^	2009	*J Bone Joint Surg Br*	Prospective	71	Arthroscopy	27.6	112	40.6	55.4
Philippon^94^	2012	*Arthroscopy*	Retrospective	64	Arthroscopy	36.0	60	15.0	69.0
Philippon^95^	2007	*Knee Surg Sports Traumatol Arthrosc*	Retrospective	65	Arthroscopy	19.2	45	31.0	6.7
Philippon^96^	2012	*Arthroscopy*	Retrospective	60	Arthroscopy	37.5	153	57.0	52.9
Philippon^98^	2010	*Am J Sports Med*	Retrospective	62	Arthroscopy	24.0	28	27.0	0.0
Polesello^99^	2014	*Hip Int*	Retrospective	62	Arthroscopy	73.2	26	34.6	12.5
Polesello^100^	2009	*Rev Bras Ortop*	Retrospective	48	Arthroscopy	27.0	28	34.0	33.0
Rafols^101^	2015	*Arthroscopy*	Prospective, Randomized	84	Arthroscopy	24.0	57	Group 1, 34.2; group 2, 36.5	47.4
Rego^102^	2018	*Int Orthop*	Retrospective	62	Arthroscopy	44.0	102	34.0	47.0
					Open	76.0	96	31.0	40.0
Rhee^103^	2016	*Arch Orthop Trauma Surg*	Prospective, Randomized	85	Arthroscopy	Group A, 32.3; group B, 31.8	37 (group A, 19; group B, 18)	Group A, 33.8; group B, 34.6	59.5
Roos^104^	2017	*Rev Bras Ortop*	Retrospective	60	Arthroscopy	29.1	41	36.1	13.0
					Open	52.0	17	35.8	31.3
Sanders^105^	2017	*Knee Surg Sports Traumatol Arthrosc*	Retrospective	65	Arthroscopy	30.0	46	42.4	67.4
Sansone^107^	2015	*Orthop J Sports Med*	Retrospective	72	Arthroscopy	12.3	115	25.0	18.0
Sansone^108^	2017	*Sc J Med Sci Sports*	Prospective	77	Arthroscopy	25.4	359	37.0	34.3
Sansone^109^	2016	*J Hip Pres Surg*	Prospective	74	Arthroscopy	12.8	80	47.0	23.0
Singh^112^	2010	*Arthroscopy*	Prospective	66	Arthroscopy	22.0	27	22.0	
Sink^113^	2013	*Clin Orthop Relat Res*	Retrospective	61	Open	27.0	52	16.2	84.1
Skendzel^114^	2014	*Am J Sports Med*	Retrospective	63	Arthroscopy	73.0	383	37.0	
							63	46.0	
Skowronek^115^	2017	*Indian J Orthop*	Retrospective	66	Open	45.0	39	29.3	35.9
Stake^116^	2013	*Am J Sports Med*	Prospective	61	Arthroscopy	24.0	42 (WC, 21; control, 21)	39.0	WC, 15; control, 15
Steppacher^117^	2014	*Clin Orthop Relat Res*	Retrospective	70	Open	72.0	97	32.0	43.0
Tjong^118^	2016	*Orthop J Sports Med*	Retrospective	46	Arthroscopy	24.0	23	Return, 44; not return, 43.7	Return, 47; not return, 53
Tjong^119^	2017	*Arthroscopy*	Prospective	62	Open	37.2	106	38.1	58.0
Tran^120^	2013	*ANZ J Surg*	Retrospective	61	Arthroscopy	14.0	41	15.7	14.7
Wang^121^	2011	*Orthop Surg*	Retrospective	51	Arthroscopy	11.6	21	37.1	57.1
Wu^122^	2019	*J Orthop Surg Res*	Retrospective	55	Mini-open	44.0	39	43.6	47.2
Zingg^123^	2013	*Arch Orthop Trauma Surg*	Prospective	68	Arthroscopy	12.0	23	27.6	21.7
					Open	12.0	15	28.9	26.7

a BD, borderline dysplastic; CLT, complete labral tear; CMS, Coleman Methodology Score; IFL, iliopsoas fractional lengthening; PRP, platelet-rich plasma; WC, workers’ compensation.

### Patient Demographics

Data from 9357 procedures (8897 patients) were collected. The median follow-up was 30.9 months (interquartile range, 24.0-45.0). The mean ± SD age was 33.4 ± 9.3 years; mean BMI, 24.8 ± 4.8; percentage right side, 55.8% ± 8.0%; and percentage female sex, 47.5% ± 20.4%. Baseline characteristics are shown in Table 1.

### Outcomes of Interest

**Figure 2.**
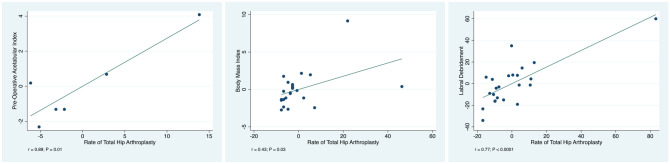
Added-variable plots of the associations: acetabular index, body mass index, and labral debridement.

The overall rate of revision was 5.29% (351 of 6641 patients), while the rate of subsequent progression to THA was 3.78% (263 of 6966 patients). Labral debridement showed evidence of a statistically significant positive and strong association with an increased rate of progression to THA (*r* = 0.77; *P* < .0001). Equally, a higher preoperative acetabular index showed evidence of a statistically significant positive and strong association with an increased rate of progression to THA (*r* = 0.89; *P* = .01). The BMI at baseline showed evidence of a statistically significant positive and moderate association with an increased rate of THA (*r* = 0.43; *P* = .03). No other statistically significant associations were found between patient characteristics, type of lesion, type of intervention, radiographic parameters, physical examination, or pre- and postoperative scores and the rate of revision and/or progression to THA. The added-variable plots of these regressions are shown in Figure 2. The multivariate analysis including all the endpoints is shown in Appendix Table A1 (available in the online version of this article).

## Discussion

FAI is a frequent and well-recognized cause for hip pain, joint damage, and early-onset osteoarthritis in young adults and athletes. Over the past few decades, better understanding of the pathophysiology and natural course of FAI has led to earlier identification and improved treatment options for this condition. The present study described the rates of revision and THA progression after surgical treatment of FAI, identifying some variables associated with increased rates of progression to THA. The overall revision rate after surgical treatment of FAI was 5.29%, and 3.78% of 9357 procedures progressed to THA. To date, this is the largest systematic review to analyze revision and progression rates after surgical treatment of FAI, including a total of 99 studies. According to the main results of the present study, BMI at baseline, labral debridement, and acetabular index were significantly associated with an increased rate of progression to THA. No other statistically significant associations were identified between patient characteristics, type of lesion, type of intervention, radiographic parameters, physical examination, or pre- and postoperative scores and the rate of revision and/or progression to THA.

Surgical hip dislocation for the treatment of FAI—including labral repair, labral debridement, femoral osteochondroplasty, and acetabuloplasty—leads to improvements in hip range of motion, radiographic parameters, and clinical outcomes comparable with those of hip arthroscopy.^30,80,91,117^ Surgical treatment for FAI, regardless of the technique, improves hip function, with 68% to 96% of patients reporting good to excellent results after a minimum follow-up of 2 years.^21^

One of the main findings of our study was an overall revision rate of 5.29% after surgical treatment of FAI (open hip dislocation, mini-open, and arthroscopic). These findings are similar to those from a registry study from the United Kingdom, which showed a revision hip arthroscopy rate of 4.5% at a mean 1.7 years.^71^ In a systematic review of >6000 patients, the reoperation rate was 6.3% at a mean 1.6 years, and the most common reason for revision surgery was progression to THA.^47^

No or mild hip osteoarthritis, labral repair, young age, and limited cartilage damage have been associated with good clinical outcomes, with a progression to THA in 0% to 26% of the cases.^21^ A systematic review compared outcomes and rates of progression to THA between surgical hip dislocation and arthroscopy^86^: 7% of the hips were converted to a THA after a maximum follow-up of 12 years in the open group, as compared with 9.5% after 8.1 years in the arthroscopic group, with no statistical difference between them. Byrd and Jones^14,15^ reported THA progression rates between 0% and 29% at 2 years after hip arthroscopy. Schairer et al^110^ used population-level data of State Ambulatory Surgery Databases and State Inpatient Databases for California and Florida from 2005 to 2012 to examine the progression rate of THA within 2 years after hip arthroscopy. They found an overall progression rate of 12.4% within 2 years after hip arthroscopy, with a significant difference between age groups. In patients <40 years old, the progression rate to THA was 3.0%, which is comparable with our findings of a 3.78% progression rate in patients with a mean age of 33.9 years. The rate of THA progression decreased steadily over time from 14.3% in 2005 to 10.3% in 2010.^110^

Age seems to be a risk factor for THA progression: patients aged >50 years exhibited a progression rate of about 20%.^71,110^ This contrasted with the findings of the present systematic review, where age was not significantly associated with a higher rate of THA progression. Differences between our results and the findings of others might be explained by the fact that we included all types of surgical treatment for FAI, instead of focusing on arthroscopic procedures; other potential reasons include the type of data used, the type of analysis conducted (registry vs systematic), and the younger age of the patients in our study.

BMI at baseline was significantly associated with an increase in the rate of THA progression at a mean follow-up of 38 months. These findings confirm previous results, which found that obesity is an independent risk factor for THA progression after hip arthroscopy at a mean follow-up of 2 years.^110^ In addition, Gupta et al^43^ and Collins et al^24^ confirmed, in small case series studies, that obesity is associated with higher rates of THA progression after arthroscopic procedures. Our results showed that BMI was a risk factor for THA progression, regardless of the surgical technique.

We were also able to show that the preoperative acetabular index was significantly associated with an increased rate of progression to THA. So far, no studies showed an association between the preoperative acetabular index and the progression rate to THA. However, high lateral center-edge angles and low acetabular indices, which require more complex surgical techniques for adequate treatment, are associated with higher rates of revision surgery.^59^

Furthermore, we found that labral debridement was associated with an increase in the rate of THA revision for the 3 major surgical techniques analyzed in the present investigation. Schilders et al^111^ demonstrated superior outcomes after labral repair as compared with labral debridement in 96 patients with a mean follow-up of 2 years. This was confirmed by Larson et al^65^ in a case-control study, with better Harris Hip Score, SF-12, and visual analog scale outcomes in the labral repair group. Menge et al^75^ compared 79 patients who underwent labral repair and 75 patients who underwent labral debridement at a mean follow-up of 10 years: no difference in clinical outcomes between the techniques was evident. However, when controlling for acetabular microfracture, Menge et al reported that labral debridement was associated with a significantly higher risk of progression to THA, confirming our findings.

This study presents several limitations. Although we have carefully followed recommended guidelines for the preparation of systematic reviews, the overall quality of the studies was low. Most of the studies were retrospective, and eligibility criteria and rehabilitation protocols were not frequently reported. The overall CMS of 64 shows acceptable quality. The mean follow-up of the studies was 30 months, which is longer than most previous studies, but revision and progression rates are likely to increase with long-term follow-up. Given these premises, the risk of biased results is moderate to high; thus, data from the present study must be interpreted with caution. The purpose of the present study was to investigate whether the aforementioned endpoints are associated with the rate of revision and subsequent progression to THA. Thus, every endpoint was investigated independently, and its risk of recurrence in revision and progression to THA was assessed. We did not perform any comparison between endpoints and their overall effect on the surgical outcomes. This may represent a limitation of the present study. However, in the current literature, there is a multitude of studies focusing on several aspects of FAI correction, evaluating and comparing all the endpoints and their effect on the surgical outcomes. Future studies should overcome these limitations, and high-quality investigations with longer follow-up should be performed.

## Conclusion

Surgical treatment for FAI leads to satisfactory outcomes. In this systematic review of 99 studies and 9357 procedures, we found an overall revision rate of 5.29% after surgical treatment. After a median follow-up of 30 months, the progression rate to THA was 3.78%. Patients were at higher risk for a subsequent progression to THA if they had a high BMI, a pathologic acetabular index, or labral debridement during correction of FAI. Therefore, we do advocate additional education of this patient population in terms of its expected outcomes and surgical labral repair instead of simple debridement if needed and technically feasible.

## Supplemental Material

sj-pdf-1-ajs-10.1177_03635465211011744 – Supplemental material for Revision Surgery and Progression to Total Hip Arthroplasty After Surgical Correction of Femoroacetabular Impingement: A Systematic Review Click here for additional data file. Supplemental material, sj-pdf-1-ajs-10.1177_03635465211011744 for Revision Surgery and Progression to Total Hip Arthroplasty After Surgical Correction of Femoroacetabular Impingement: A Systematic Review by Filippo Migliorini, Nicola Maffulli, Alice Baroncini, Jörg Eschweiler, Markus Tingart and Marcel Betsch in The American Journal of Sports Medicine
