# Supplementary material for: Revision Surgery and Progression to Total Hip Arthroplasty After Surgical Correction of Femoroacetabular Impingement: A Systematic Review
Source: Am J Sports Med. 2021 Jun 3;50(4):1146–56. doi: 10.1177/03635465211011744 (PMC8980457; doi:10.1177/03635465211011744)
Supplement: sj-pdf-1-ajs-10.1177_03635465211011744 – Supplemental material for Revision Surgery and Progression to Total Hip Arthroplasty After Surgical Correction of Femoroacetabular Impingement: A Systematic Review [file sj-pdf-1-ajs-10.1177_03635465211011744.pdf]

Appendix Table A1. Overall results of the multivariate analysis

Endpoint	Revision Surgeries			Total Hip Arthroplasty		
	**Observations**	***r***	***P***	**Observations**	***r***	***P***
Mean Age	36	-0.14	0.4	44	0.26	0.1
Female sex	32	0.12	0.5	37	0.06	0.7
BMI	25	0.00	1.0	23	0.43	0.03
Right Side	12	0.42	0.2	14	-0.04	0.9
Labral debridement	6	-0.56	0.2	23	0.77	< 0.0001
Labral repair	8	-0.24	0.6	30	0.11	0.6
Labral reconstruction	2	-1.00	1.0	11	-0.03	0.9
Capsular repair	0			11	-0.57	0.1
Acetabular index pre-op	2	1.00	1.0	6	0.89	0.01
Acetabular index post-op	2	1.00	1.0	5	-0.60	0.3
Caudocranial femoral coverage post-op	0			3	0.73	0.5
Anterior femoral coverage pre-op	0			3	-0.93	0.2
Anterior femoral coverage post-op	0			3	0.99	0.1
Posterior femoral coverage pre-op	0			3	-0.92	0.3
Posterior femoral coverage post-op	0			3	-0.98	0.1
Medial joint space pre-op	0			4	-0.15	0.8
Foveal joint space pre-op	0			2	1.00	1.0
Lateral joint space pre-op	0			5	0.19	0.8
Femoral offset pre-op	0			3	0.77	0.4
Femoral offset post-op	0			0		
VAS pre-op	11	-0.24	0.5	14	0.31	0.3
VAS post-op	11	-0.14	0.7	14	0.12	0.7
Tönnis pre-op	12	-0.26	0.4	9	-0.25	0.5
Tönnis post-op	5	0.69	0.2	3	0.98	0.1
Alfa angle pre-op	20	-0.18	0.5	25	-0.10	0.6
Alfa angle post-op	10	-0.33	0.3	14	0.24	0.4
Gamma angle pre-op						
Gamma angle post-op						
Sharp angle post-op	0			3	0.42	0.7
CEA pre-op	4	-0.44	0.6	7	0.35	0.4
CEA post-op	0			0		
aCEA pre-op	5	0.18	0.8	5	0.12	0.9
aCEA post-op	0			0		
lCEA pre-op	20	-0.01	1.0	18	-0.05	0.8
lCEA post-op	5	-0.54	0.3	4	-0.90	0.1
Flexion pre-op	11	0.43	0.2	11	-0.07	0.8
Flexion post-op	4	-0.71	0.3	2	-1.00	1.0
Extention pre-op	0			0		
Extention post-op	0			0		
Abduction pre-op	7	0.08	0.9	8	-0.37	0.4
Abduction post-op	0			0		
Adduction pre-op	2	1.00	0.0	3	-0.99	0.1
Adduction post-op	0			0		

Internal rotation pre-op	11	0.25	0.5	12	0.23	0.5
Internal rotation post-op	4	0.89	0.1	4	0.74	0.3
External rotation pre-op	11	0.34	0.3	10	-0.16	0.7
External rotation post-op	4	-0.04	1.0	2	-1.00	1.0
Anterior impingement test pre-op	6	-0.35	0.5	5	0.41	0.5
Anterior impingement test post-op	0			0		
Lateral impingement test pre-op	3	-0.35	0.8	3	0.67	0.5
Lateral impingement test post-op	0			0		
Posterior impingement test pre-op	3	-0.20	0.9	3	0.09	0.9
Posterior impingement test post-op	0			0		
Cross sign pre-op	5	0.57	0.3	5	-0.20	0.7
Cross sign post-op	2	1.00	1.0	0		
Tönnis 0 pre-op	12	0.05	0.9	17	0.08	0.8
Tönnis I pre-op	11	-0.12	0.7	16	0.02	0.9
Tönnis II pre-op	4	-0.84	0.2	8	-0.02	1.0
Tönnis 0 post-op	2	1.00	1.0	2	-1.00	1.0
Tönnis I post-op	2	1.00	1.0	3	0.39	0.7
Tönnis II post-op	2	-1.00	1.0	3	0.99	0.1
Harris Hip Score pre-op	28	0.10	0.6	30	-0.27	0.1
Harris Hip Score post-op	30	-0.17	0.4	33	-0.14	0.4
NAHS pre-op	16	0.13	0.6	17	-0.05	0.9
NAHS post-op	16	0.18	0.5	17	-0.14	0.6
SF-36 Physical pre-op	5	-0.09	0.9	6	-0.40	0.4
SF-36 Physical post-op	5	-0.34	0.6	6	-0.54	0.3
SF-36 Mental pre-op	5	-0.39	0.5	5	0.94	0.2
SF-36 Mental post-op	5	-0.26	0.7	5	0.47	0.4
HOS ADL pre-op	19	0.05	0.8	13	0.15	0.6
HOS ADL post-op	19	0.05	0.8	13	-0.61	0.3
HOS-SSS pre-op	24	0.00	1.0	18	-0.16	0.5
HOS-SSS post-op	26	0.09	0.7	20	-0.37	0.1
iHOT 12 pre-op	0			0		
iHOT 12 post-op	4	-0.07	0.9	5	-0.24	0.7
Return to sport	3	-0.90	0.3	2	1.00	1.0
